# What drives successful verbal communication?

**DOI:** 10.3389/fnhum.2013.00622

**Published:** 2013-10-01

**Authors:** Miriam de Boer, Ivan Toni, Roel M. Willems

**Affiliations:** ^1^Donders Institute for Brain, Cognition and Behaviour, Radboud University NijmegenNijmegen, Netherlands; ^2^Max Planck Institute for PsycholinguisticsNijmegen, Netherlands

**Keywords:** communication, language, individual differences, mentalizing, Raven’s progressive matrices

## Abstract

There is a vast amount of potential mappings between behaviors and intentions in communication: a behavior can indicate a multitude of different intentions, and the same intention can be communicated with a variety of behaviors. Humans routinely solve these many-to-many referential problems when producing utterances for an Addressee. This ability might rely on social cognitive skills, for instance, the ability to manipulate unobservable summary variables to disambiguate ambiguous behavior of other agents (“mentalizing”) and the drive to invest resources into changing and understanding the mental state of other agents (“communicative motivation”). Alternatively, the ambiguities of verbal communicative interactions might be solved by general-purpose cognitive abilities that process cues that are incidentally associated with the communicative interaction. In this study, we assess these possibilities by testing which cognitive traits account for communicative success during a verbal referential task. Cognitive traits were assessed with psychometric scores quantifying motivation, mentalizing abilities, and general-purpose cognitive abilities, taxing abstract visuo-spatial abilities. Communicative abilities of participants were assessed by using an on-line interactive task that required a speaker to verbally convey a concept to an Addressee. The communicative success of the utterances was quantified by measuring how frequently a number of Evaluators would infer the correct concept. Speakers with high motivational and general-purpose cognitive abilities generated utterances that were more easily interpreted. These findings extend to the domain of verbal communication the notion that motivational and cognitive factors influence the human ability to rapidly converge on shared communicative innovations.

## INTRODUCTION

Daily human communication is surprisingly effective, even though it involves producing and understanding utterances that are inherently ambiguous. The potential mapping between behavior and intentions in communication is very large and many-to-many, such that similar behaviors can indicate different intentions and vice versa.

The ability of humans to map behavior to intentions has been labeled *interactive intelligence *(Levinson, 2006) and might be supported by motivational factors and cognitive abilities. The cognitive abilities implicated in understanding the intentions, feelings or thoughts of others, are often labeled as Theory of Mind or mentalizing abilities (Premack and Woodruff, 1978; Baron-Cohen and Wheelwright, 2004; Frith and Frith, 2012). Motivational factors refer to the drive to invest resources to understand another individual, the willingness and motivation to spend energy understanding the mental states of others (Levinson, 2006; Tomasello, 2009). In an alternative account, it is proposed that most of the time interlocutors would not have to infer the mental state of the other’s mind at all. Automatic alignment of representations of the other’s message-meaning mapping by tight coupling of production and comprehension (Pickering and Garrod, 2004) or the many cues generated during interaction (Shintel and Keysar, 2009) would suffice. Under most circumstances, no specific mentalizing skills would be needed to solve the many-to-many mapping problem. In this perspective, communicative coordination relies on general-purpose cognitive abilities, as if communication would be similar to complex problem solving. The latter account gets credibility from the finding that, considering the speed of human communication, mentalizing as the only strategy to solve the multi-mapping problem is implausible as it would require extensive cognitive and temporal resources (Shintel and Keysar, 2009; Lin et al., 2010).

Here, we test whether motivational factors, mentalizing abilities, or general cognitive abilities in speakers predict successful tailoring of a message in a verbal communication game. For instance, an agent might have extremely sophisticated computational abilities and be able to store/retrieve a very large set of behavior/meaning mappings, but fail to do anything if not motivated to communicate, or fail to adjust a sophisticated behavior/meaning mapping to an Addressee and make it comprehensible. Different cognitive abilities involved in human communication might be differently sensitive to the expression of psychological traits across a group of individuals (Baron-Cohen et al., 2005; De Ruiter et al., 2010). Individual variation can help us understand the general principles of human communication (Levinson and Gray, 2012). In this study we investigate which psychometric scores indexing motivational factors and cognitive abilities, contribute most to a Communicator’s success.

Previous research investigated individual sources of variation in subject pairs engaged in a non-verbal communication game (Volman et al., 2012). The design in that study focussed on how pairs of Communicators establish communicative strategies, and how inter-subject differences influence communicative success. Communicators’ motivation to solve complex tasks, as indexed by the Need for Cognition Scale (NCS; Cacioppo et al., 1996), predicted communicative success. General intelligence of the Addressees, as indexed by the Raven’s advanced progressive matrices (RAPM; Raven et al., 1995) accounted for higher accuracy scores. Although attribution of mental states to another person (mentalizing) seems an important capacity for creating a new communication system that both Communicator and Addressee can comprehend, the speed and success with which such a new communicative system was established could not be explained by the participants’ score on the empathy quotient (EQ; Baron-Cohen and Wheelwright, 2004), or a similar measure for empathy, the interpersonal reactivity index (IRI; Davis, 1983). In a related study using the same non-verbal communication game, the magnitude of communicative *adjustments* to a presumed Addressee *was* explained by the EQ (Newman-Norlund et al., 2009). Senders high in empathy put greater emphasis on crucial communicative elements when they believed their Addressee was a child compared to when they believed their Addressee was an adult. In contrast, individuals with high motivation for complex problems (NCS; Cacioppo et al., 1996) were less likely to adapt their communicative behavior toward their Addressee.

The picture that emerges from those studies on non-verbal communication systems is that empathic traits may be beneficial for *adapting* communicative behavior to another individual. In contrast, the ability to *generate* effective communicative acts might be mainly influenced by the motivation and ability to solve complex problems.

Here, we tested the role of trait variables on the ability to generate successful communicative interaction in the verbal domain by indexing individual differences in empathizing (IRI and EQ, respectively Davis, 1983; Baron-Cohen and Wheelwright, 2004) Need for Cognition Scale (NCS, Cacioppo et al., 1984), general intelligence (RAPM, Raven et al., 1995) and verbal intelligence (Groninger Intelligentie Test Matrix Reasoning, and the Wexler Adult Intelligence Scale Similarity and Vocabulary subscale, respectively Kooreman and Luteijn, 1987; WAIS-III, 1997). Abstract visuo-spatial abilities were indexed as part of the RAPM (Carpenter et al., 1990; Mackintosh and Bennett, 2005). We will examine how these factors in the Communicator contribute to successful communication, that is, generating an accurate and easy interpretable message for an Addressee (see Ickes et al., 2000 on the role of motivation on empathic accuracy in observators) in the context of an interactive word game.

**FIGURE 1:**
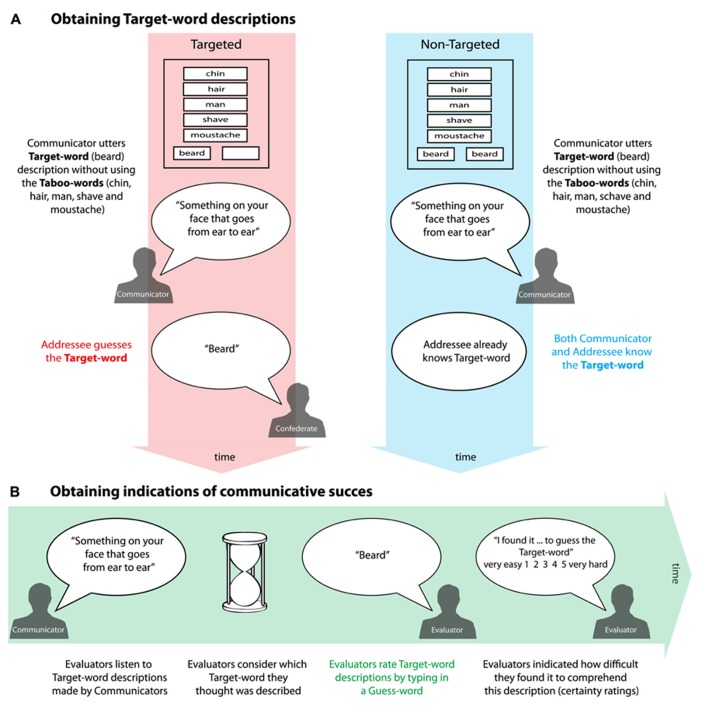
**(A)** Time line of the Taboo game. In an fMRI experiment Communicators had to describe a Target-word (“beard”) to an Addressee (confederate) without using Taboo-words (“hair,” “chin,” “man,” “shave” and “mostache”). In the TARGETED setting (depicted in red), Communicators were made to believe that the Addressee was unbeknownst of the Target-word (right empty box next to the Target-word “beard”). In the NON- TARGETED setting (depicted in blue), Communicators were aware that the Addressee already knew the Target-word. Communicators were reminded of this by printing the Target-word twice on the Communicator’s screen. **(B)** Obtaining indications of communicative success (in green). First, Evaluators, naive of the Taboo game experiment, listened to the Target- word description made during the Taboo game. Second, Evaluators were asked to consider which Target-word they thought was described. Third, they were to type in their answer (Guess-word) and lastly, they filled out how difficult they found it to come up with their answer on a scale from one till five (1 “easy,” 5 “difficult”; certainty scores). A measure of Communicators’ success was obtained by counting the Evaluators’ correct guesses divided by the total amount of trials per condition.

In the interactive word game, both communicative setting and linguistic difficulty were independently manipulated. We used a paradigm called the Taboo game (Willems et al., 2010) where a Communicator had to describe a Target-word (e.g., “Beard”) to an Addressee in one sentence without using Taboo-words (e.g., “man,” “shave,” “hair,” “chin” and “mostache”; see **Figure 1A**). An indication of the Target-word description’s communicative success was obtained by evaluation of these utterances by a new group of subjects (labeled as Evaluators, see **Figure 1B**). The data reported in this manuscript relates the performance of these Evaluators to the psychometric scores of Communicators. We predict to find a similar pattern as described above: not mentalizing abilities *per se*, but the motivation or general cognitive ability to solve complex tasks will account for effective communication in an existing verbal communication system. This study aims to open the way for understanding variations in visual perspective-taking abilities during social interactions. Accordingly, we pay particular attention to the RAPM as an index of visuo-spatial abilities (Carpenter et al., 1990; Mackintosh and Bennett, 2005).

## MATERIAL AND METHODS

### SUBJECTS

Sixteen participants (labeled as Communicators, four males, mean age = 21 years old, SD = 3 years) played the Taboo game in the context of an fMRI experiment (for further details, see Willems et al., 2010) and completed several psychometric tests. All had Dutch as their mother-tongue, and did not have a known neurological history, hearing problems, dyslexia, stuttering or other language-related problems. In a separate experimental session, sixteen subjects naive to the Taboo game evaluated the Target-word descriptions generated by the Communicators. These Evaluators (four males, mean age = 20 years old, SD = 3 years) did not have language, hearing or eyesight difficulties and had Dutch as their mother tongue. The data reported in this manuscript relates the performance of the Evaluators to the psychometric scores of Communicators.

### PROCEDURE

#### Description from communicators

Experimental material was obtained in the context of an fMRI study (for further details, see Willems et al., 2010). Communicators generated descriptions for a confederate (referred to as Addressee) after which we obtained their psychometric scores on various cognitive abilities and motivational factors (for details of the acquisition of the Communicators’ psychometric scores, see Psychometric indexes of individual cognitive abilities of Communicators). In a separate study, a group of new participants labeled as Evaluators rated these descriptions’ communicative success.

Communicators made descriptions of 60 concrete nouns (Target-words). They would for instance have to describe the Target-word “beard” without using five so called Taboo-words “hair,” “chin,” “man,” “shave” and “mustache” (see **Figure 1A**). Communicator and Addressee could clearly hear each other’s utterances via MR (Magnetic Resonance) compatible headphones, with the Addressee inferring the Target-word that the Communicator described. Since the Communicator was lying in the MR scanner, we filtered out scanner noise using the audacity noise reduction function (Audacity from http://audacity.sourceforge.net/) to increase the audibility of the Target-word descriptions. Descriptions lasted on average 5.14 s (SD = 0.68 s). In the Taboo game, two factors were manipulated: communicative setting and linguistic difficulty. Communicative setting was manipulated by changing the Communicator’s belief of the Addressee’s knowledge of the Target-word. In the TARGETED setting the Communicator generated the description for a specific other (a confederate), who gave wrong answers on a prescribed set of trials (30% of the trials). In case of a wrong trial, Communicators were asked to generate a new Target-word description consecutively. These repeated trials were not rated by the Evaluators. In the NON-TARGETED setting, it was explained to Communicators that the Addressee was already aware of the Target-word and that this person was only overhearing the Communicator’s Target-word description. Communicators were reminded that the Addressee already knew the Target-word by printing the Target-word twice on the Communicator’s screen (see **Figure 1A**). Linguistic difficulty was manipulated by varying the semantic distance between Target-word and Taboo-words. During EASY trials, Communicators described Target-words without using Taboo-words that were loosely semantically related to the Target-word (e.g., Target-word “rainbow,” Taboo-words: “four-leaf-clover,” “violet,” “water,” “sound,” “fairy-tale”). During DIFFICULT trials, Communicators described Target-words without using Taboo-words that were closely semantically related to the Target-word (such as the “beard” example above).

During the TARGETED and the NON-TARGETED setting, half of the trials were EASY, and half of the trials were DIFFICULT. Lexical frequency of Taboo and Target-words was matched between all conditions (CELEX database, Baayen et al., 1995). Stimulus lists were pseudo-randomized in two sets such that participants did not describe the same Target-words in TARGETED and NON-TARGETED trials. Half of the Communicators described Target-words of set A in the TARGETED setting and Target-words of set B in the NON-TARGETED setting. The other half of the Communicators described Target-words in the opposite settings, meaning set B in the TARGETED setting and set A in the NON-TARGETED setting. More Communicators completed Set A during the TARGETED setting. To prevent Evaluators from hearing certain Target-word descriptions more often generated in the TARGETED or the NON-TARGETED setting, four out of the twenty Communicators of the original Taboo game experiment were excluded at random. With sixty Target-word descriptions of sixteen Communicators; there were a total of 960 unique Target-word descriptions.

#### Evaluators

In the current experiment, a new group of subjects evaluated these Target-word descriptions from the Willems et al. (2010) study to obtain an indication of the Communicator’s communicative success. After reading a written instruction, Evaluators completed three practice trials not used in the remainder of the experiment, and then performed the actual task in two blocks of approximately thirty minutes each. Trials were separated in different phases (see **Figure 1B**). At first, a black screen was presented in which a fixation cross appeared. The Evaluators heard a Target-word description made by one of the Communicators, e.g., “Something on your face that goes from ear to ear.” Evaluators planned their response with a cut-off time of twenty seconds and typed which Target-word they thought was described (Guess-word). Thereafter, Evaluators were asked to give a score from one to five on how difficult they found it to generate their answer with “1” meaning that they found this very difficult and “5” meaning that they found this very easy (from now on referred to as “certainty score”). After a randomized intertrial interval (mean = 4.5 s, SD = 0.93 s), the next trial was presented. The experiment was performed using Presentation software (Version 10.2, www.neurobs.com) and presented on a laptop computer via earphones. Stimulus presentation was pseudo-randomized such that each Communicator’s Target-word description was rated by two different Evaluators. In total, each Evaluator heard a total of 120 unique Target-word descriptions, eight from the same Communicator: two recorded during the TARGETED EASY condition, two recorded during the TARGETD DIFFICULT condition, two during the NON-TARGETED EASY and two during the NON-TARGETED DIFFICULT condition. Descriptions of the same Communicator or the same Target-word would never be presented in immediate succession; neither would Evaluators hear a description of a particular Target-word more than once per block. For instance, in the first block, Evaluators would hear a recording of a Target-word description of “beard” by Communicator A, and in the second block they would hear a recording of a Target-word description of “beard” by Communicator B.

#### Psychometric indexes of individual cognitive abilities of Communicators

After playing the Taboo game, each Communicator completed psychometric tests to characterize their empathizing abilities (IRI and EQ, respectively Davis, 1983; Baron-Cohen and Wheelwright, 2004), motivation for complex tasks (NCS, Cacioppo et al., 1984), general intelligence (RAPM, Raven et al., 1995) and verbal intelligence (GIT matrices, WAIS Similarity and WAIS Vocabulary subscale, respectively Kooreman and Luteijn, 1987; WAIS-III, 1997). Since the focus of our paper was on the Communicator, no psychometric indexes of cognitive abilities or motivational factors were taken from the Evaluators.

The EQ indexes both cognitive and affective empathy. It characterizes cognitive empathy (mentalizing), reactivity and social skill but is not correlated with social desirability (Baron-Cohen and Wheelwright, 2004; Lawrence et al., 2004). Instead of calculating one scale, empathy can also be indexed in four subscales as is done in the IRI (Davis, 1983). The Perspective Taking subscale indexes the ease with which one can take the point of view or perspective of the other. The Fantasy subscale indexes how easily somebody can identify himself/herself with a fictional character. There are two subscales of emotional reactions: the Empathic Concern subscale indexes feelings of compassion and warmth, while the Personal Distress subscale indexes the tendency to feel discomfort when observing another person in distress. Motivation to be engaged in complex tasks, such as we assume the Taboo game is, was indexed with the NCS (Cacioppo et al., 1984). The EQ, IRI and NCS are self-report Likert scale type questionnaires. All three questionnaires were completed with paper and pencil.

Raven’s advanced progressive matrices (Raven et al., 1995) index general intelligence. Two separate factors underlie performance on the RAPM. Part of the items are solved by verbal-analytical rules, whereas other items tend to be solved using visual-spatial rules (Carpenter et al., 1990; DeShon et al., 1995). Communicators had to complete as many of the 36 items (RAPM set II) as possible within twenty minutes. The Communicator’s RAPM score was calculated by adding up the number of correctly completed items within that time.

Communicators high in verbal intelligence may have a larger vocabulary and, due to their increased word reasoning skills, have easier access to alternatives for Taboo-words. The WAIS Vocabulary subscale (WAIS-III, 1997) indexes word understanding and how well this word understanding can be expressed. Participants are asked to give definitions of words that become increasingly more unfamiliar. Word reasoning skills were indexed by the Groninger Intelligence Test Matrix Reasoning subscale (GIT Matrix Reasoning, Kooreman and Luteijn, 1987). Participants are asked to solve analogies, such as “if table is to wood, stove is to iron, thus shoe is to⋯” During the WAIS Similarity subscale (WAIS-III, 1997), participants are asked to describe how common objects or concepts are similar, e.g., “what is the similarity between a bike and a car?” All the verbal intelligence subscales were taken orally and scored according to prescribed standards (Kooreman and Luteijn, 1987; WAIS-III, 1997).

#### Communicative success

Our measure of communicative success was based on the correct guesses of the Evaluators divided by the total amount of trials per condition. In the following cases, we rated the Evaluators’ guesses as correct: if the Guess-word had exactly the same word form as the Taboo word, if the Guess-word was a compound instead of a head, or vice versa (for example “woonwijk” or “wijk” meaning “living district” and “neighborhood”), if it was a synonym (“leunstoel” by “fauteuil,” meaning “armchair” and “lounge chair”), or if it was a diminutive (e.g., “munt” by “muntje” meaning “coin” and “little coin”). In this manner, we were able to consider successful communication of word *meaning*.

### STATISTICAL ANALYSIS

Accuracy and certainty scores of Evaluators were analyzed using a 2 × 2 within subjects ANOVA with factors setting (TARGETED and NON-TARGETED) and linguistic difficulty (EASY and DIFFICULT). First, to assess which psychometric indexes explained variance in description quality, we performed a regression analysis with communicative success in the TARGETED setting as a dependent variable. Second, to correct for the individual differences in general performance on the Taboo game, a second analysis was conducted comparing the TARGETED to the NON-TARGETED setting by subtracting the communicative success scores obtained from the TARGETED and the NON-TARGETED setting. Third, regression analyses were conducted to investigate which cognitive traits explained communicative success during our manipulation of linguistic difficulty (DIFFICULT, EASY and EASY subtracted from DIFFICULT). In each regression analysis, the Communicators’ psychometric scores on all tests were entered as independent regressors in a stepwise fashion: a variation on the forward algorithm. Only those independent factors whose contribution was unique and significant were entered in the model (*p* < 0.05), while at each subsequent search step redundant factors were removed. Since questionnaires indexing the same cognitive ability may potentially correlate, e.g., mentalizing ability was indexed by both the EQ and the IRI), we considered whether predictors correlated strongly with one another, but Pearson’s correlation coefficients were <0.8 across regressors. Only independent variables explaining unique variance are reported. All statistical analyses were conducted with IBM SPSS Statistics for Windows (Version 19.0).

## RESULTS

### REACTION TIMES, CERTAINTY RATINGS AND ACCURACY SCORES

Evaluators on average took 2.5 s (SD = 0.5 s) to generate a Guess-word. Evaluators found the task rather difficult (mean certainty rating = 2.25, SD = 0.29, 1–5 scale). However, Evaluators comprehended the Communicators’ Target-word descriptions well (mean percentage correct = 73%, SD = 5%, minimum score 62% and maximum 83%). There was no interaction in reaction times, certainty ratings, or accuracy scores between communicative setting (TARGETED, NON-TARGETED) and difficulty (EASY, DIFFICULT), neither was there a main effect of setting (TARGETED, NON-TARGETED). Evaluators planned shorter, were more certain and more accurate for Target-word descriptions made in the EASY condition (for statistics see **Table 1**).

**Table 1:** Repeated measures analysis of variance was applied on reaction times, certainty ratings and accuracy scores of Evaluators when listening to Target-word descriptions made by Communicators in an earlier conducted fMRI experiment.

	F(df)	MSe	p
**Reaction times**			
Communication	<1 (1,15)	32.2	0.73
Linguistic difficulty	11.25 (1,15)	30.66	<0.01
Communication × linguistic difficulty	1.48 (1,15)	53.37	0.24
**Certainty ratings**			
Communication	2.78 (1,15)	0.05	0.12
Linguistic difficulty	11.75 (1,15)	0.06	<0.01
Communication × linguistic difficulty	1.53 (1,15)	0.07	0.24
**Accuracy**			
Communication	<1 (1,15)	0.02	0.87
Linguistic difficulty	7.45 (1,15)	0	<0.05
Communication × linguistic difficulty	<1 (1,15)	0.01	0.91

*The model contained the factors communicative setting (descriptions that Evaluators listened to were made in the TARGETED or the NON-TARGETED setting) and linguistic difficulty (descriptions were made in the EASY or DIFFICULT condition). Evaluators planned shorter (*F*(1,15) = 11.25, *p* < 0.01), were more certain (*F*(1,15) = 11.75, *p* < 0.01) and more accurate (*F*(1,15) = 7.45, *p* < 0.05) for Target-word description made in the easy condition.
*

### COMMUNICATIVE SUCCESS AND INDIVIDUAL DIFFERENCES

**FIGURE 2:**
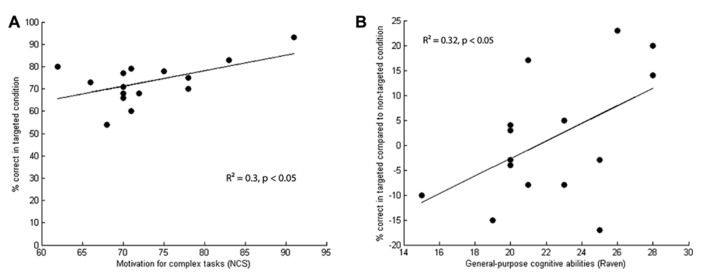
**Communicative success as evaluated by a new group of participants (in percentage correct) plotted against the psychometric indexes of the Communicators. (A)** Communicators’ scores on motivation for complex tasks as indexed by the NCS (*R*^2^ = 0.3, *p* < 0.05, regression line is solid, data points represented as dots) drive communicative success in the communicative setting (TARGETED condition). **(B)** Communicators’ scores on general intelligence as indexed by Raven’s Advanced Progressive Matrices (RAPM, *R*^2^ = 0.32, *p* < 0.05, regression line is solid, data points represented as closed dots) drive communicative success in the TARGETED setting compared to the NON-TARGETED setting. A positive difference score indicates that Communicators performed better in the TARGETED setting, a negative score that Communicators performed better in the NON-TARGETED setting. To correct for individual differences in general performance on the Taboo game, a model to account for communicative success during the targeted setting compared with the NON-TARGETED setting was created. The difference in accuracy scores between the two conditions was positively driven by the Communicator’s general intelligence as indexed by the Raven’s APM. Neither the EQ, nor any of the IRI subscales could account for the difference in success across the communicative settings.

Only those regressors explaining a statistically significant portion of variance are described here (for statistics see **Table 2**). Communicative success during the TARGETED setting was positively driven by the Communicators’ motivation to solve complex tasks as indexed by the NCS (**Table 2**, see **Figure 2A**). No such effect was observed during the NON-TARGETED trials. Indexes of empathy (IRI, EQ) did not account significantly for variance in performance.

**Table 2:** Overview of psychometric indexes significantly accounting for communicative success in the different experimental conditions.

Experimental condition	Psychometric index	Beta	*F*(df)	*R*^2^	*p*
Targeted	NCS	0.54	5.86 (1,14)	0.3	<0.05
Targeted - non-targeted	Raven’s APM	0.56	6.43 (1,14)	0.32	<0.05
Difficult	WAIS vocabulary	0.65	9.98 (2,13)	0.61	<0.01
	IRI personal distress	0.46			
Easy	NCS	0.55	8.4 (2,13)	0.56	<0.05
	IRI personal distress	0.51			

To correct for individual differences in general performance on the Taboo game, a model to account for communicative success during the TARGETED setting compared with the NON-TARGETED setting was created. The difference in accuracy scores between the two conditions was positively driven by the Communicator’s general intelligence as indexed by the Raven’s APM (see **Table 2**, **Figure 2B**). Neither the EQ, nor any of the IRI subscales could account for the difference in success across the communicative settings.

Verbal abilities as indexed with the WAIS vocabulary subscale positively accounted for communicative success during DIFFICULT trials (collapsed across TARGETED and NON-TARGETED settings). Furthermore, the Communicator’s score on the IRI personal distress subscale, which indexes the tendency to feel discomfort when observing somebody else’s distress, was predictive of accuracy scores on DIFFICULT trials. For EASY trials, the same subscale (IRI personal distress) and the Communicator’s NCS positively accounted for communicative success. None of the psychometric indexes explained variance of communicative success in DIFFICULT *compared to* EASY trials. 

## DISCUSSION

We have employed inter-subject differences in trait parameters and communicative performance to examine whether motivational factors, mentalizing skills, or general-purpose cognitive abilities preferentially accounted for communicative success. In an interactive verbal communication task, participants (Communicators) were asked to describe concepts without using a number of semantically related words (Willems et al., 2010). Successful communication was quantified by how frequently a group of new participants (Evaluators) would infer the correct concept. We found that motivational factors, as indexed by the Communicator’s motivation to solve complex tasks (NCS), were positively driving successful communication in a communicative (“TARGETED”) setting. These findings extend previous observations (Volman et al., 2012) to the domain of verbal communication, to show the importance of motivational factors in communicative behavior. Communicators high in need for cognition may make more effort to select the message/meaning mapping that is best comprehensible. They may be more flexible in finding alternatives, if the solution they generated turned out to be incomprehensible for their Addressee (Cacioppo et al., 1984; Evans et al., 2003). However, need for cognition did not explain variance in communicative success, when we directly compared the TARGETED versus the NON-TARGETED settings. That is, need for cognition was important in explaining performance during the communicative (TARGETED) trials overall, but not when directly comparing TARGETED versus NON-TARGETED trials. Comparing TARGETED versus NON-TARGETED settings directly revealed that communicative success was significantly predicted by Communicators’ general-purpose cognitive ability as indexed by Raven’s APM (Raven et al., 1995). A Communicator’s high general intelligence may be beneficial for the generation of efficient messages in several ways. It may help storage of speaker history (Horton and Gerrig, 2005; Shintel and Keysar, 2009; Galati and Brennan, 2010), executive control (Ybarra and Winkielman, 2012), and working memory capacity (Lin et al., 2010). This idea fits with recent evidence showing tightly matched neural dynamics in subjects solving communicative and rule-based solo problems (Stolk et al., 2013).

From our findings, we can only speculate as to whether Communicator’s success in this communication game is driven by general cognitive abilities, or more specifically by visuo-spatial abilities. Research on the underlying cognitive processes of the RAPM has suggested that some of Raven’s matrices are solved using a visuo-spatial strategy (Carpenter et al., 1990; DeShon et al., 1995) for an alternative view see (Plaisted et al., 2011). This abstract visuo-spatial ability may positively drive effective search of alternatives for words that cannot be used to generate the Target-word description (Taboo-words). Communicators with a high RAPM score may be more skilful in finding words that can be easily interpreted by the Addressee, and as a consequence, be more effective in solving the message-to-meaning problem.

Given that the communication task used in this study relied on verbal material, it might appear surprising that the psychometric indexes of verbal ability (GIT or WAIS subscales, Kooreman and Luteijn, 1987; WAIS-III, 1997) did not significantly account for variation in communicative success. Yet, the verbal intelligence of the Communicator (WAIS) *was* important for solving trials where the Taboo-words were closely semantically related to the Target-word (DIFFICULT trials). This may be an indication that linguistic abilities accounted for communicative success in semantically difficult trials in general, but not for communicative trials specifically. These findings support the notion of a cognitive difference between linguistic and communicative abilities (Willems and Varley, 2010; Willems et al., 2011).

Importantly, mentalizing abilities, as indexed by general cognitive empathy, emotional reactivity, social skill (EQ, Lawrence et al., 2004) or as indexed by the Perspective Taking, Fantasy, Empathic Concern and Personal Distress subscales (IRI; Davis, 1983), were also not significantly related to communicative success as a function of the communicative setting. Yet, a Communicator’s personal distress was important for solving trials where Taboo-words were closely semantically related to the Target-word (DIFFICULT trials). This result is not immediately compatible with the idea that mentalizing abilities are important for generating a comprehensible message. However, this does not preclude the possibility that mentalizing abilities are important for implementing communicative *adjustments* toward a specific Addressee, as previously shown in the context of non-verbal communication (Newman-Norlund et al., 2009). Nor does it preclude that mentalizing abilities are employed in communicative task settings. As a matter of fact, the fMRI data of the study from which our materials were taken, shows that participants activate mentalizing related brain areas when designing a communicative message for a specific other (Willems et al., 2010). The present findings add to this that the *individual differences *in mentalizing abilities are not indicative of communicative success, but this obviously does not mean that such abilities are not used in communication.

The current study is a first step in the direction to point out the role of motivational factors and cognitive abilities on verbal communicative success. Given that the main experiment was performed in an MR environment, the interaction was quite rigidly structured and, as a consequence, not all constituents of social interaction (De Jaegher et al., 2010; Schilbach et al., 2012) were present during the game. For instance, the role of Communicator and Addressee was fixed, and there was a maximum duration of the time interval during which Communicator and Addressee were allowed to speak. Our task *was* interactive in the sense that Communicators were actively engaged in our verbal interaction game. Interlocutors’ performance depended on the clarity of the description of the Communicator and the comprehension of the Addressee. The interlocutors could to some extent monitor and adjust their behavior on the basis of feedback (correct or incorrect), and on the timing of the on-line interaction (e.g., time interval required by a Communicator to organize an utterance, and by an Addressee to reply). In this study, we focussed on the role of the Communicator. Future research should study the effect of cognitive abilities and motivational factors on *both* interlocutors and should investigate additional factors that could be of influence on communicative success, such as the role of motivation to engage in social interaction or the extent of the pre-existing common ground (e.g., strangers or close friends). Not only should these factors be studied at the individual level, but also on the “second person” level, the level that comes about *between* interactors (Becchio et al., 2010; De Jaegher et al., 2010; Schilbach et al., 2012).

More generally, our data speak to the observation that if a Communicator has a global idea of her Addressee, she may not always need to employ mentalizing abilities immediately or exclusively (Shintel and Keysar, 2009). As Zaki and Ochsner (2012) put it, in communication it is not *either* mentalizing, *or* general cognitive abilities, but more a question of “when/how” the one system is used and when/how the other system is used.

## CONCLUSION

We have employed individual variation to examine whether motivational factors, mentalizing skills, or general cognitive abilities preferentially accounted for communicative success. We found that motivational factors (“need for cognition”) and general-purpose cognitive abilities (Raven’s matrices) were positively driving successful communication in an interactive communication task. These findings extend previous observations (Volman et al., 2012) to the domain of verbal communication and stress the importance of motivation and general-purpose cognitive abilities in communicative success. Mentalizing or empathy scores did not explain communicative success in the paradigm that we employed here. Future research should be directed toward understanding under which circumstances communicative behavior is most driven by motivational and general cognitive factors, and when differences in mentalizing abilities between individuals do make a difference.

## Conflict of Interest Statement

The authors declare that the research was conducted in the absence of any commercial or financial relationships that could be construed as a potential conflict of interest.
